# Effect of tonsillectomy combined with steroid pulse therapy upon IgA nephropathy depending on proteinuria status at diagnosis: a nationwide multicenter cohort study in Japan

**DOI:** 10.1007/s10157-024-02530-6

**Published:** 2024-07-02

**Authors:** Hiroyuki Komatsu, Shouichi Fujimoto, Yuji Sato, Takashi Yasuda, Yoshinari Yasuda, Keiichi Matsuzaki, Keita Hirano, Tetsuya Kawamura, Takashi Yokoo, Yusuke Suzuki, Shoichi Maruyama

**Affiliations:** 1 Center for Medical Education and Career Development, Faculty of Medicine, University of Miyazaki, 5200 Kihara, Kiyotake, Miyazaki, 889-1692 Japan; 2 Department of Medical Environment Innovation, Faculty of Medicine, University of Miyazaki, Miyazaki, Japan; 3 Department of Internal Medicine, Division of Nephrology, National Health Insurance Takachiho Town Hospital, Takachiho, Miyazaki, Japan; 4 Department of Internal Medicine, Kichijoji Asahi Hospital, Tokyo, Japan; 5 Department of Nephrology/CKD Initiatives, Nagoya University Graduate School of Medicine, Nagoya, Japan; 6 Department of Public Health, Kitasato University School of Medicine, Kanagawa, Japan; 7 Division of Nephrology, Department of Internal Medicine, Japanese Red Cross Ashikaga Hospital, Ashikaga, Japan; 8 Division of Nephrology and Hypertension, Department of Internal Medicine, Jikei University School of Medicine, Tokyo, Japan; 9 Division of Nephrology, Department of Internal Medicine, Faculty of Medicine, Juntendo University, Tokyo, Japan; 10 Division of Nephrology, Department of Internal Medicine, Faculty of Medicine, University of Nagoya, Nagoya, Japan

**Keywords:** IgA nephropathy, Glomerulonephritis, Tonsillectomy, Steroid pulse therapy, Proteinuria, Kidney outcome

## Abstract

**Background:**

The effects of tonsillectomy combined with steroid pulse (TSP) therapy for IgA nephropathy (IgAN) are little known. Therefore, we examined the effects of TSP therapy on the kidney outcomes of IgAN in a large, nationwide cohort study in Japan.

**Methods:**

Between 2002 and 2004, 632 IgAN patients with ≥ 0.5 g/day proteinuria at diagnosis were divided into three groups with mild (0.50–0.99 g/day; *n* = 264), moderate (1.00–1.99 g/day, *n* = 216), or severe (≥ 2.00 g/day; *n* = 153). Decline in kidney function and urinary remission were compared among the three groups after TSP therapy, corticosteroid (ST) therapy, or conservative therapy during a mean follow-up of 6.2 ± 3.3 years. 10.6% and 5.9% of patients in the ST and conservative therapy group underwent tonsillectomy.

**Results:**

The rate of urinary remission at the final observation was significantly higher in the TSP therapy group than in the ST or conservative therapy groups (mild proteinuria: 64%, 43%, and 41%; moderate proteinuria: 51%, 45%, and 28%; severe proteinuria: 48%, 30%, and 22%, respectively). In contrast, the rate of a 50% increase in serum creatinine was lower in groups TSP therapy, than ST or conservative therapy (mild proteinuria: 2.1%, 10.1% and 16.7%; moderate proteinuria: 4.8%, 8.8% and 27.7%; severe proteinuria: 12.0%, 28.9% and 43.1%, respectively). In multivariate analysis, TSP therapy significantly prevented a 50% increase in serum creatinine levels compared with conservative therapy in groups with moderate and severe proteinuria (hazard ratio, 0.12 and 0.22, respectively).

**Conclusion:**

TSP significantly increased the rate of proteinuria disappearance and urinary remission in IgAN patients with mild-to-moderate urinary protein levels. It may also reduce the decline in kidney function in patients with moderate-to-severe urinary protein levels.

## Introduction

IgA nephropathy (IgAN) is still the most frequent glomerular disease in the world since its initial report in 1969 [1, 2]. Even in Japan Renal Biopsy Registry (J-RBR) data, IgAN is the most common, accounting for about 30% of diagnosis by kidney biopsy [3, 4]. In Japan, the prognosis of IgAN is poor [5], with approximately 50% of patients developing kidney failure after 30 years [6]. The involvement of galactose-deficient IgA 1 (Gd-IgA1) molecules has been postulated as a cause of this disease, and the development and widespread use of specific treatments for this disease are expected [7].

The Kidney Disease Improving Global Outcomes (KDIGO) guidelines published in 2021 [8] emphasize the importance of blood pressure (BP) control and the administration of the maximum dose of renin–angiotensin system blockade (RAS-B) in the treatment of IgAN. They also stated the importance of discussing the individual risks and benefits of corticosteroid therapy. In addition, tonsillectomy can be considered for a specific population of Japanese patients.

In Japan, the combination of tonsillectomy and steroid pulse (TSP) therapy has been aggressively implemented since 2000 [9–20]. The palatine tonsil is assumed to be a site of an abnormal mucosal immune response in IgAN [21–23], based on the idea that removal of the palatine tonsil as a lesion is effective. Moreover, tonsillectomy is expected to have a synergistic effect with corticosteroid therapy to suppress the inflammation induced by Gd-IgA1 [24]. However, few studies have enrolled a sufficient number of patients to determine in which disease severity the therapeutic effect is best exerted.

In the present secondary study, we examined whether TSP was more effective than corticosteroid or conservative treatment in a nationwide multicenter retrospective cohort study [25] already conducted in Japan. In this study, we focused on differences in the amount of proteinuria at diagnosis as an indicator of disease severity. The efficacy of treatment was also examined not only in terms of remission in urinary findings but also in terms of worsening kidney function and end-stage kidney disease (ESKD).

## Methods

### Study design and participant selection

This study was conducted as a secondary study of a Japanese nationwide multicenter retrospective cohort study [25] conducted by the Ministry of Health, Labor and Welfare’s Research Group on Intractable Renal Diseases. The administrative office for this study was established at the St. Marianna Medical University, and the study plan was reviewed and approved by the university’s Ethics Committee (IRB No. 2101). Ethical reviews were conducted and approved by each collaborating institution (e.g., University of Miyazaki, IRB No. 2015-016). This study was conducted in compliance with the principles of the Declaration of Helsinki.

The study population consisted of 1,174 patients aged 18 years or older diagnosed with IgAN by percutaneous kidney biopsy at 42 cooperating institutions nationwide from 2002 to 2004. The last follow-up date for this study was January 31, 2014. Anonymized data with urinary findings or kidney function deficits at diagnosis and the last observation were excluded [25]. The present study focused on proteinuria at diagnosis as an indicator of disease severity. Patients with urinary protein levels less than 0.5 g/day at diagnosis (*n* = 446) were excluded for a separate study of prognostic factors [26]. Finally, 632 patients were included in this study.

**Fig. 1:**
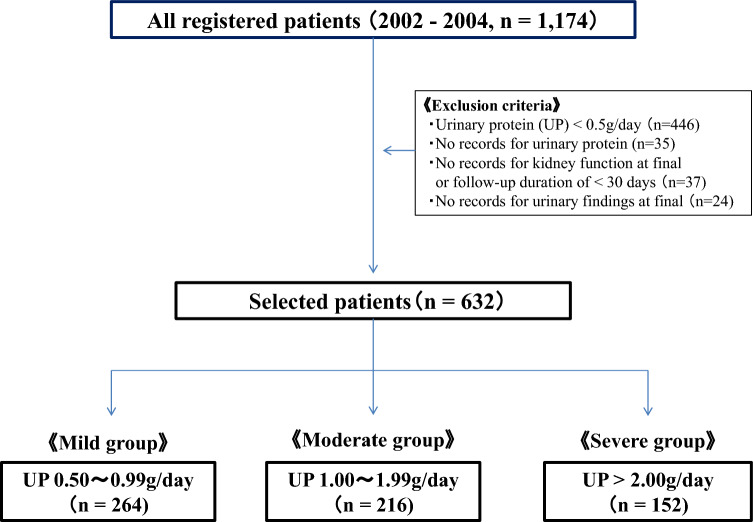
Flowchart of the study design. UP: urinary protein

Based on the severity of urinary protein levels, patients were divided into three treatment groups: mild (0.50–0.99 g/day, *n* = 264), moderate (1.00–1.99 g/day, *n* = 216), and severe (≥ 2.00 g/day, *n* = 152). The mean observation period for patients in the current study was 6.2 ± 3.3 years. Figure 1 shows a flowchart of the study design.

### Evaluation of clinical findings

Data on age, sex, body mass index (BMI), BP, urinary dip-stick findings and sediment, 24-h proteinuria, urinary protein–creatinine ratio (UP/UCr), estimated GFR (mL/min/1.73 m^2^) and serological findings of creatinine (sCr), blood urea nitrogen (BUN), uric acid, albumin, total cholesterol, triglyceride, IgA, and C3 at diagnosis and initial treatment were collected from medical records. Information on kidney function (sCr, eGFR) and serological and urinary findings was collected every 3 months during follow-up and final observation. Hypertension was defined as systolic BP > 140 mmHg and/or diastolic BP > 90 mmHg, or use of antihypertensive drugs before diagnosis. The qualitative findings of urinary protein (UP) and urinary occult blood (UOB) were each scored as follows: − and ± as 0; 1+ as 1; 2+ as 2; 3+ as 3. The severity of the histological findings was arbitrarily collected at the discretion of each institution.

### Classification of initial treatments

In the nationwide multicenter retrospective cohort study [25], initial treatment (initiated within 1 year after kidney biopsy) was categorized by tonsillectomy and corticosteroid therapy into six categories: tonsillectomy and corticosteroids with pulse therapy (T1S2), tonsillectomy and corticosteroids without pulse therapy (T1S1), tonsillectomy without corticosteroids (T1S0), corticosteroids with pulse therapy (T0S2), corticosteroids without pulse therapy (T0S1), and conservative therapy such as RAS-B without tonsillectomy and corticosteroids (T0S0). In this study, the initial treatment was divided into three groups: TSP therapy (T1S2; *n* = 114), corticosteroid (ST) therapy (T1S1; *n* = 26, T0S2; *n* = 102, and T0S1; *n* = 118), and conservative therapy (T1S0; *n* = 16, T0S0; *n* = 256). In the conservative therapy group, RAS-B was administered in 196 (72.1%) patients and tonsillectomy was performed in 16 (5.9%) patients. The treatment efficacy of the TSP group was compared with that of the other two treatment groups.

### Definition of clinical remission and study outcomes

The primary outcome was a decline in kidney function with a 50% increase in sCr from baseline or ESKD with dialysis initiation and preemptive kidney transplantation. The secondary outcome was clinical remission (CR), which was defined as the disappearance of hematuria and proteinuria. The disappearance of hematuria was defined as < 5/HPF of red blood cells in sediment or (–) or (±) in the dip-stick test. The disappearance of proteinuria was also defined as < 0.3 g/day of protein in 24-h urine, a UP/UCr ratio of < 0.3 in spot urine or (–) or (±) in the dip-stick test.

### Statistical analysis

All continuous variables are presented as means ± standard deviation (SD). The clinical parameters of the three groups were compared using single-factor analysis of variance (ANOVA) for normally distributed continuous variables or the Kruskal–Wallis test for non-normally distributed continuous variables. Cumulative probabilities of 50% increase in sCr from baseline or ESKD with dialysis initiation were analyzed using the Kaplan–Meier method, and differences in curves were compared using the log-rank test. The effect of multiple covariates on the rate of the primary endpoints was assessed using a Cox proportional hazards model. All independent variables used in multivariate analyses were either categorical (coded as 0/1) or quantitative. Treatments with TSP and ST therapy were considered categorical variables. Age, BMI, systolic BP, eGFR, serum albumin, uric acid, and total cholesterol levels were quantitative variables. The results of the multivariate analysis were expressed as hazard ratios (HR) for 50% increase in sCr from baseline with 95% confidence intervals (CI). A *p* value of < 0.05 was considered significant for all data, which were statistically analyzed using IBM SPSS Advance Statistical Version 27.0.

## Results

### Clinical findings at diagnosis and initial treatment

Table 1 shows the basic background information of the patients at diagnosis and initial treatment. The mean UP values in the mild, moderate, and severe groups were 0.71, 1.37, and 3.45 g/day, respectively. As the severity of proteinuria increased, the BP, sCr, and serum uric acid levels increased significantly, whereas the eGFR and serum albumin levels decreased significantly. During the initial treatment, the TSP percentage did not change significantly and ranged from 16.4 to 19.4% among the three groups. However, as the severity of proteinuria increased, the percentage of patients receiving ST therapy increased and the percentage receiving conservative therapy decreased. The percentage of patients who received RAS-B increased significantly from 56.8 to 75.7% as the severity of proteinuria increased.

**Table 1:** Baseline characteristics of groups before treatment (*n* = 632)

		Mild-UP	Moderate UP	Severe UP	*P* value
		(*n* = 264)	(*n* = 216)	(*n* = 152)	
	Age (years)	38.6 ± 14.5	40.3 ± 15.2	43.9 ± 16.4	0.003*
	Sex (M/F)	126 / 138	109 / 107	84 / 68	0.334
	Body Mass Index	22.6 ± 3.45	22.8 ± 3.80	23.2 ± 3.49	0.249
	Systolic BP (mmHg)	126.8 ± 17.8	127.2 ± 19.1	133.5 ± 18.6	0.001*
	Diastolic BP (mmHg)	76.5 ± 12.5	76.7 ± 12.5	78.4 ± 12.4	0.308
	Proteinuria (g/day)	0.71 ± 0.14	1.37 ± 0.29	3.45 ± 1.77	< 0.001*
	Urine occult blood > 3+ (*n*, %)	141 (53.4%)	134 (62.0%)	99 (65.1%)	0.084
	Serum creatinine (mg/dL)	0.90 ± 0.37	0.97 ± 0.39	1.26 ± 0.90	< 0.001*
	Estimated GFR (mL/min/1.73 m^2^)	76.5 ± 26.0	71.2 ± 28.9	60.6 ± 27.6	< 0.001*
	Blood urea nitrogen (mg/dL)	15.6 ± 5.26	16.4 ± 5.74	19.6 ± 7.56	< 0.001*
	Serum uric acid (mg/dL)	5.89 ± 1.49	6.03 ± 1.63	6.59 ± 1.61	< 0.001*
	Serum total protein (g/dL)	6.81 ± 0.50	6.59 ± 0.66	6.02 ± 0.72	< 0.001*
	Serum albumin (g/dL)	4.02 ± 0.40	3.81 ± 0.44	3.38 ± 0.64	< 0.001*
	Serum total cholesterol (mg/dL)	196.1 ± 37.6	211.2 ± 42.1	235.3 ± 72.2	< 0.001*
	Serum IgA (mg/dL)	345.0 ± 127.1	357.7 ± 141.4	333.5 ± 131.5	0.257
	Serum C3 (mg/dL)	101.8 ± 21.3	103.2 ± 22.4	107.2 ± 27.5	0.101
	*Initial treatment*				< 0.001*
	Tonsillectomy and steroid pulse	47 (17.8%)	42 (19.4%)	25 (16.4%)	
	Corticosteroid therapy	79 (29.9%)	91 (42.1%)	76 (50.0%)	
	Conservative therapy	138 (52.3%)	83 (38.4%)	51 (33.6%)	
	Concomitant use of RAS-B	150 (56.8%)	138 (63.9%)	115 (75.7%)	0.001*

*RAS-B* renin–angiotensin system blockade

^*^ANOVA, Chi-square independent test or Pearson’s Chi-square test

### Remission rates of urinary findings at final observation

**Fig. 2:**
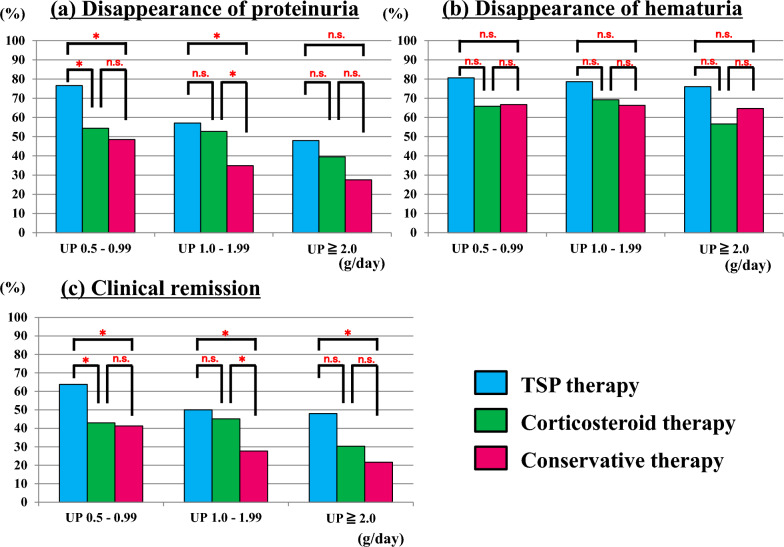
Comparison of urinary findings at final observation. *TSP* tonsillectomy combined with steroid pulse therapy, *UP* urinary protein. *n.s.* not significant, *p < 0.05

Figure 2 shows the percentage of disappearance of UP and UOB and remission of urinary findings (CR) at the last observation in each group according to the treatment modality. The percentage of UP disappearance was significantly higher in all severity groups, in the order of TSP, ST, and conservative therapy (mild proteinuria, 76.6% vs. 54.4% vs. 48.5%; moderate proteinuria, 57.1% vs. 52.7% vs. 34.9%; and severe proteinuria, 48.0% vs. 39.5% vs. 27.5%, respectively). However, the percentage of UOB disappearance did not differ between the treatment modalities. The number of patients with negative UOB before treatment was 4 (3.5%), 3 (1.2%), and 36 (13.2%) in the TSP, ST, and conservative therapy groups, respectively (*p* < 0.001). The percentage of CR was significantly higher with TSP therapy than with ST or conservative therapy (mild proteinuria, 63.8% vs. 43.0% vs. 41.3%; moderate proteinuria, 50.5% vs. 45.1% vs. 27.7%; and severe proteinuria, 48.0% vs. 30.3% vs. 21.6%, respectively).

### Kidney function at final observation

**Fig. 3:**
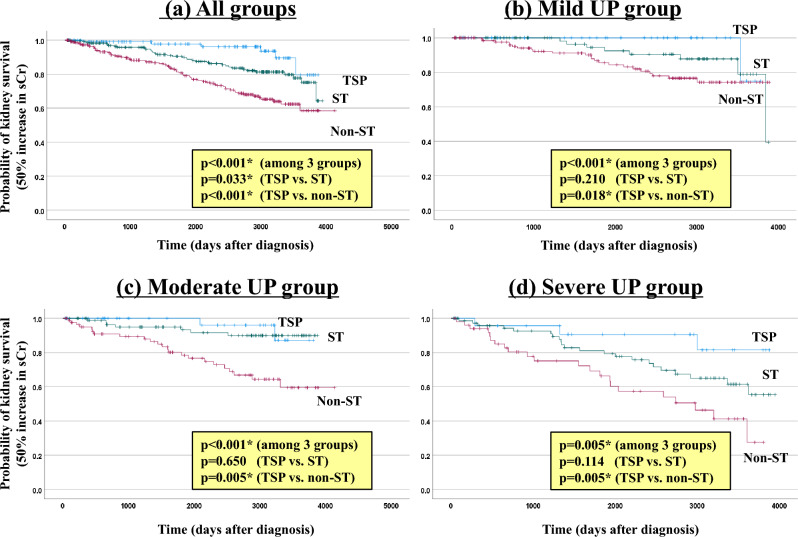
Comparison of prognosis (50% increase in sCr) among treatment modalities. Statistical analysis was performed using the log-rank test for Kaplan–Meier curves. *TSP* tonsillectomy combined with steroid pulse therapy, *ST* corticosteroid therapy, *UP* urinary protein, *sCr* serum creatinine

**Fig. 4:**
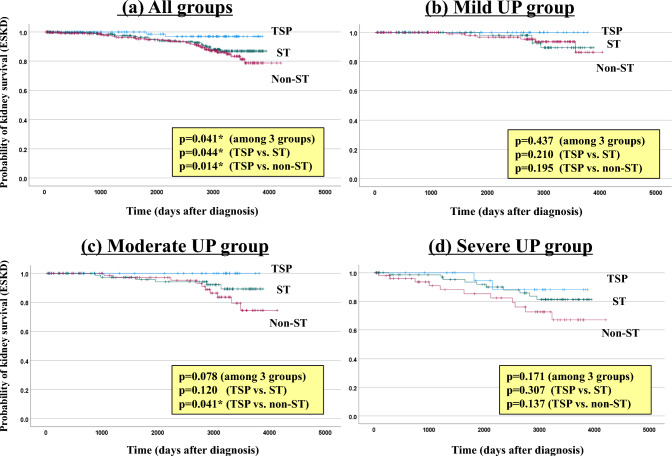
Comparison of prognosis (progression to ESKD) among treatment modalities. Statistical analysis was performed using the log-rank test for Kaplan–Meier curves. *TSP* tonsillectomy combined with steroid pulse therapy, *ST* corticosteroid therapy, *UP* urinary protein, *ESKD* end-stage kidney disease

Of all patients, 112 (17.7%) had a 50% increase in sCr from baseline at the last observation. Based on severity, 32 (12.1%), 33 (15.3%), and 47 (30.9%) patients in the mild, moderate, and severe groups, respectively, achieved a 50% increase in sCr. TSP, ST, and conservative therapy led to a 50% increase in sCr in 6 (5.3%), 38 (15.4%), and 68 (25.0%) patients, respectively. The rate of a 50% increase in sCr was lower in groups TSP therapy, than ST or conservative therapy (mild proteinuria: 2.1%, 10.1% and 16.7%; moderate proteinuria: 4.8%, 8.8% and 27.7%; severe proteinuria: 12.0%, 28.9% and 43.1%, respectively). In addition, 49 (7.8%) patients developed ESKD (39 patients with hemodialysis initiation, 9 patients with peritoneal dialysis initiation, one patient with preemptive kidney transplantation) at the last observation. TSP, ST, and conservative therapy led to ESKD in 2 (1.8%), 20 (8.1%), and 27 (9.9%) patients, respectively, and this outcome was significantly less common with TSP than the other treatments (*p* < 0.001, Chi-square test). Figures 3 and 4 show the Kaplan–Meier curves for a 50% increase in sCr and ESKD with dialysis initiation, respectively, in the urinary protein severity groups. In all patients, the 50% increase in sCr and ESKD differed significantly between the TSP, ST, and conservative therapy groups (*p* < 0.001 and *p* = 0.041 by log-rank test). In particular, in all groups, TSP significantly suppressed the 50% increase in sCr, especially when compared with conservative therapy. On the other hand, comparison of the TSP and ST groups showed no significant difference in the 50% increase in sCr and ESKD occurrence in the stratified urine protein group. The 10-year kidney survival rates were 97.0%, 87.0%, and 78.7% for TSP, ST, and conservative therapy, respectively.

### Analysis of factors contributing to renal outcome

The Cox proportional hazards model was used to evaluate the effects of clinical findings and the modality of initial treatment on the 50% increase in sCr from baseline. The major risk factors for IgAN progression, including TSP, ST, and conservative therapy, were selected as imperative independent variables in the model (Table 2). Age in the mild group; sex, BMI, eGFR, serum albumin level, and serum total cholesterol level in the moderate group; and eGFR level in the severe group were poor prognostic factors. TSP significantly reduced the decline in kidney function compared with conservative therapy in the moderate and severe groups (HR, 0.119; 95% CI 0.025–0.569 and HR, 0.221; 95% CI 0.058–0.839, respectively). In contrast, in the mild group, it had no significant effect on kidney function.

**Table 2:** Multivariate analysis of factors in a 50% increase in sCr from baseline

		Mild group (0.50–0.99 g/day) (*n* = 264)			Moderate group (1.00–1.99 g/day) (*n* = 216)			Severe group (≧ 2.00 g/day) (*n* = 152)		
	Variable	Hazard ratio	95% CI	*P*	Hazard ratio	95% CI	*P*	Hazard ratio	95% CI	*P*
	Age (/1 year)	1.071	(1.032–1.111)	< 0.001*	0.997	(0.965–1.030)	0.855	0.987	(0.966–1.009)	0.238
	Sex (male)	2.299	(0.778–6.794)	0.132	0.323	(0.110–0.948)	0.040*	1.271	(0.564–2.868)	0.563
	BMI (kg/m^2^)	0.980	(0.838–1.146)	0.803	0.860	(0.753–0.982)	0.026*	0.995	(0.895–1.106)	0.921
	Systolic BP (/1 mmHg)	0.992	(0.963–1.021)	0.569	0.985	(0.961–1.009)	0.208	1.015	(0.992–1.038)	0.200
	Estimated GFR (/1 mL/min/1.73 m^2^)	1.009	(0.982–1.036)	0.524	0.965	(0.942–0.989)	0.004*	0.963	(0.944–0.982)	< 0.001*
	Serum albumin (/0.1 g/dL)	0.888	(0.287–2.745)	0.836	0.320	(0.138–0.740)	0.008*	0.644	(0.375–1.106)	0.111
	Serum uric acid (/0.1 mg/dL)	1.420	(0.920–2.191)	0.114	1.136	(0.870–1.484)	0.348	0.979	(0.720–1.331)	0.891
	Serum total cholesterol (/1 mg/dL)	0.986	(0.972–1.001)	0.059	1.013	(1.002–1.024)	0.023*	0.997	(0.992–1.002)	0.215
	Conservative therapy	1 (ref.)	–	–	1 (ref.)	–	–	1 (ref.)	–	–
	TSP therapy	0.302	(0.036–2.517)	0.268	0.119	(0.025–0.569)	0.008*	0.221	(0.058–0.839)	0.027*
	Corticosteroid therapy	0.391	(0.106–1.447)	0.159	0.145	(0.051–0.416)	< 0.001*	0.497	(0.228–1.083)	0.079

^*^Statistically significant

### Adverse events of each treatment

65 adverse events were observed in 47 patients in this study: 5 events in the TSP therapy group, 42 in the ST therapy group, and 18 in the conservative therapy group. Infections occurred in eight cases, including four cases of pneumonia, in five patients in the ST-treated group, but none in the TSP group. Diabetes occurred in 8 patients in the ST therapy group. In the TSP therapy group, one patient of gastric ulcer, one patient of pancreatitis, and three patients of complications related to tonsillectomy occurred, but no serious adverse events were observed.

## Discussion

The primary nationwide multicenter cohort study [25] analyzed 1,065 of 1,174 registered patients with IgAN. The group that underwent tonsillectomy (*n* = 252) was compared with the group that did not undergo tonsillectomy (*n* = 813). The primary outcome (50% increase in sCr from baseline or ESKD) was significantly reduced in the tonsillectomy group (HR, 0.43; 95% CI, 0.20–0.85), which was similar after 1:1 propensity score matching and inverse probability of treatment weighted estimation (IPTW) matching. The rate of additional treatment within 1 year of diagnosis was also significantly lower (HR, 0.37; 95% CI, 0.20–0.63). This study included 632 patients with urinary protein levels of 0.5 g/day or higher, and treatment efficacy was compared between TSP therapy (T1S2), ST therapy (T1S1 and T0S2 and T0S1), and conservative therapy (T1S0 and T0S0). The CR rate was significantly higher in the TSP group than in the conservative therapy group, regardless of the urinary protein level at diagnosis. The incidence of the primary outcome (a 50% increase in sCr from baseline or ESKD with dialysis initiation) was also significantly reduced.

Studies comparing TSP with other treatments using the percentage of CR in IgAN as an outcome have been conducted mainly in Japan [9–20]. These results show an association between the proteinuria severity before treatment and the CR rate after treatment. Concretely, at pretreatment proteinuria levels of 0.5–1.0 g/day, 1.0–1.5 g/day, and 1.5–2.0 g/day, proteinuria disappeared at estimated rates of 70%, 60%, and 50%, respectively. However, most of these were small, single-center cohort studies. The present study was based on a large multicenter cohort, and the percentages of disappearance of proteinuria with TSP in the mild (0.50–0.99 g/day), moderate (1.00–1.99 g/day), and severe groups (≥ 2.00 g/day) were 76.6%, 57.1%, and 48.0%, respectively. These results are consistent with those of previous studies.

There have been only a few reports on the effect of TSP on IgAN in reducing kidney function decline [20, 27–29]. These studies reported that TSP significantly reduced kidney function decline compared with other treatments; however, outcome measures varied, including ESKD, doubling of sCr, and a 25% decrease in eGFR. Hoshino et al. [29], in a study of 1,127 patients with IgAN at four hospitals, found that when UP levels were 1.0 g/day or higher, TSP was significantly more effective than RAS-B treatment in suppressing the ESKD progression. In contrast, in patients with UP levels less than 1.0 g/day, there was no difference in the efficacy between TSP, corticosteroid therapy, and RAS-B. This is consistent with the results of the current study, in which the primary outcome was a 50% increase in sCr levels from baseline or dialysis initiation. The effect of TSP on kidney function decline in the mild group could not be statistically demonstrated in multivariate analysis. A nationwide secondary cohort study of patients with urinary protein < 0.5 g/day at diagnosis [26] also found no effect of tonsillectomy or corticosteroid therapy on reducing kidney function decline. This may be partly due to the small number of primary endpoints that occurred in patients with mild IgAN during the limited observation period.

A large prospective cohort study was also conducted in Japan (Japan IgA Nephropathy Prospective Cohort Study: J-IGACS) [30], in which 1,130 patients with IgAN were enrolled between 2005 and 2015. Finally, 941 patients were included in the analysis, and the efficacy of treatment was compared among three groups: corticosteroid plus tonsillectomy, corticosteroid therapy, and non-corticosteroid therapy. Corticosteroid therapy and corticosteroid plus tonsillectomy significantly reduced primary outcomes (50% increase in sCr from baseline or dialysis initiation) compared with non-corticosteroid therapy. Furthermore, corticosteroid plus tonsillectomy reduced the rate of the primary outcomes significantly more than corticosteroid therapy (HR 0.40, 95% CI 0.18–0.91).

These results suggest that TSP may lead to better CR in mild IgAN. In contrast, if performed in patients with moderate-to-severe IgAN, it is expected to preserve kidney function over the medium to long term.

Abnormalities in the mucosal–bone axis may be involved in the pathogenesis of IgAN [31]. This indicates an increased production of Gd-IgA1 in IgAN in the early stages according to the multi-hit theory [32], which is a tentative theory of the pathogenesis of IgAN. Whether the gut-associated lymphoid tissue (GALT) or nasal-associated lymphoid tissue (NALT) is the primary site of mucosal immune abnormalities is debatable. The efficacy of intestinal-selective corticosteroids in the NEFIGAN trial [33] indicates that GALT may be important in the pathogenesis of IgAN. Meanwhile, the production of Gd-IgA1 from tonsil cells in patients with IgAN [21] and the decrease in serum Gd-IgA1 levels after tonsillectomy [22] suggested that NALT may contribute to the production of Gd-IgA1. Tonsillectomy may prevent the progression and relapse of IgAN by eliminating abnormal sites of mucosal immunity. Liu et al. also reported the benefit of tonsillectomy for CR and suppression of ESKD progression in a meta-analysis of 14 studies [34]. In addition, in the primary nationwide multicenter cohort study, tonsillectomy was relatively safe, with complications occurring in only seven of 252 patients (2.8%) [25]. Recently, a series of therapies specific to the pathogenesis of IgAN have been developed, such as anti-B cell and anti-plasmacytoid therapies targeting Gd-IgA1 and regulators of complement activity [7]. In the future, these drugs will become the mainstay of treatment; however, until then, tonsillectomy will be an effective treatment for eliminating Gd-IgA1.

The current study has several limitations. First, although this was a large multicenter cohort study, it focused on initial treatment and did not adequately examine treatment during the course of the study. Second, the initial treatment in this study took place between 2002 and 2004, and the treatment methods, such as the percentage of RAS-B administered, may have changed. In fact, in a questionnaire survey conducted in 2021 among members of the Japanese Society of Nephrology, 88.6% of patients with IgAN with urinary protein of 1.0 g/day or more responded that they would receive RAS-B [35]. Third, this study did not consider the detailed methods of steroid pulse therapy. There are two major methods of steroid pulse therapy: the Pozzi method and the Hotta method [9, 36]. In this study, the treatment advantage of the TSP group over the ST group was very limited when analyzed stratified by urinary protein level. It is possible that this was due to differences in the modalities of steroid pulse therapy. In contrast, a secondary national multicenter cohort study reported that the number of steroid pulse courses did not affect kidney prognosis [37]. Future detailed studies are needed to consider more definite indications for TSP therapy. Fourth, in the present study, we performed a multivariate analysis of prognostic factors, stratified by severity based on the amount of proteinuria. Therefore, statistical analyses such as propensity score matching and IPTW matching were not performed, which may not have adequately controlled for confounding factors and selection bias of the initial treatment. Fifth, histopathological factors were not included in the prognostic analysis in the present study because information on histological severity was not made a mandatory registration at the study design stage. Finally, all participants in the present study were adult Japanese, and it is unclear whether similar results can be obtained in other racial groups.

In conclusion, TSP significantly increased the rate of proteinuria disappearance and CR in patients with IgAN, regardless of the amount of proteinuria at diagnosis. It may also reduce the decline in kidney function in patients with moderate-to-severe urinary protein levels.
